# Na^+^/H^+^ Exchanger Isoform 1 Induced Cardiomyocyte Hypertrophy Involves Activation of p90 Ribosomal S6 Kinase

**DOI:** 10.1371/journal.pone.0122230

**Published:** 2015-04-01

**Authors:** Maiy Jaballah, Iman A. Mohamed, Bayan Alemrayat, Fatima Al-Sulaiti, Mohamed Mlih, Fatima Mraiche

**Affiliations:** College of Pharmacy, Qatar University, Doha, Qatar; Cleveland Clinic, UNITED STATES

## Abstract

Studies using pharmacological and genetic approaches have shown that increased activity/expression of the Na^+^/H^+^ exchanger isoform 1 (NHE1) play a critical role in the pathogenesis of cardiac hypertrophy. Despite the importance of NHE1 in cardiac hypertrophy, severe cerebrovascular side effects were associated with the use of NHE1 inhibitors when administered to patients with myocardial infarctions. p90 ribosomal S6 Kinase (RSK), a downstream regulator of the mitogen-activated protein kinase pathway, has also been implicated in cardiac hypertrophy. We hypothesized that RSK plays a role in the NHE1 induced cardiomyocyte hypertrophic response. Infection of H9c2 cardiomyoblasts with the active form of the NHE1 adenovirus induced hypertrophy and was associated with an increase in the phosphorylation of RSK (*P*<0.05). Parameters of hypertrophy such as cell area, protein content and atrial natriuretic mRNA expression were significantly reduced in H9c2 cardiomyoblasts infected with active NHE1 in the presence of dominant negative RSK (DN-RSK) (*P*<0.05). These results confirm that NHE1 lies upstream of RSK. Increased phosphorylation and activation of GATA4 at Ser^261^ was correlated with increased RSK phosphorylation. This increase was reversed upon inhibition of RSK or NHE1. These findings demonstrate for the first time that the NHE1 mediated hypertrophy is accounted for by increased activation and phosphorylation of RSK, which subsequently increased the phosphorylation of GATA4; eventually activating fetal gene transcriptional machinery.

## Introduction

Cardiovascular diseases (CVDs) remain one of the leading causes of death worldwide despite the advances in treatment [1]. Cardiomyocyte hypertrophy (CH), a condition that occurs in response to mechanical load and neurohormonal stimulation, is characterized by an increase in cardiomyocyte size, enhanced protein synthesis and the reactivation of the fetal gene program [2]. Pathological CH results in left ventricular dysfunction and heart failure if left unresolved [3]. Neurohormonal stimulation mediated by α-adrenergic agonists including phenylephrine (PE), endothelin-1 and angiotensin II (ANG II) has been demonstrated to activate the Na^+^/H^+^ exchanger isoform 1 (NHE1) and contribute to hypertrophy [4, 5]. NHE1 is a ubiquitously expressed housekeeping glycoprotein, in which the activity contributes to regulating intracellular pH (pH_i_) through a 1:1 stoichiometric exchange of H^+^ for extracellular Na^+^ [6, 7]. Previous reports have suggested that increased NHE1 activity is involved in the pathogenesis of cardiac pathologies [8] including CH [9, 10] and ischemia/reperfusion (I/R) injury [11] in both *in vivo* and *in vitro* models. Complimentary genetic evidence for the key role of NHE1 in CH was further emphasized by our group, which demonstrated that transgenic mice expressing a cardiac specific active form of NHE1, rather than a wild type NHE1 form, had an exacerbated hypertrophic response [12]. Furthermore, pharmacological inhibition of NHE1 activity *in vivo* was sufficient to reduce isoproterenol-induced CH [13–15]. The implication of NHE1 in CH addresses a need to inhibit the deleterious effect of increased NHE1 activity in the heart, while salvaging its homeostatic functions [16]. Therefore, a better understanding of the molecular mechanisms downstream of the NHE1 activation contributing to CH are necessary in order to develop more specific strategies to indirectly inhibit NHE1.

Recent reports suggest that NHE1 regulates the activity of various subfamilies of the mitogen activated protein kinase (MAPKs) family in the setting of hypertrophy [17]. Both extracellular regulated kinase 1/2 (ERK1/2) and NHE1 have been demonstrated to be activated upon stimulation with ANG II in rat aortic smooth muscle cells, an effect that was attenuated by inhibition of NHE1 [18]. Similarly, EMD87580, an NHE1 inhibitor attenuated the activation of both ERK1/2 and p38 MAPK following stimulation with PE in neonatal rat ventricular cardiomyocytes (NRVMs) [19]. However, p90 ribosomal S6 kinase (RSK), a Ser/Thr protein kinase and a well known downstream effector of ERK, and one whose activity and phosphorylation levels were elevated in patients with end stage dilated cardiomyopathy [20] was not evaluated in these settings. RSK is activated upon phosphorylation at multiple residues. These phosphorylation events are triggered by ERK1/2 at Thr^573^ in the C-terminal kinase domain of RSK [21]. Interestingly, RSK has also been demonstrated to be a primary regulator of NHE1 activity through phosphorylation of Ser^703^ at the NHE1 C-terminal, and to facilitating the binding of the 14-3-3 protein to the phosphorylated Ser^703^ residue [22]. Moreover, in NRVMs and adult rat ventricular cardiomyocytes (ARVMs), RSK has been demonstrated to play a key role in the activation of ribosomal RNA synthesis in response to hypertrophic stimulation by endothelin-1 and PE [23]. It is therefore hypothesized that RSK plays an important role in the NHE1 induced cardiomyocyte hypertrophic response. The mechanism by which NHE1-regulates RSK has never been clearly defined.

GATA binding protein 4 (GATA4), a zinc finger transcription factor which acts as a crucial regulator of cardiac development and hypertrophy in response to hypertrophic PE stimulation in NRVMs has been identified as the main effector of RSK [24, 25]. RSK, specifically RSK isoform 2, was shown to activate GATA4 through phosphorylation at Ser^261^, suggesting that RSK mediates the GATA4 dependent reactivation of cardiac fetal genes [26]. The present study was conducted to determine whether RSK could account for hypertrophy induced by active NHE1 in H9c2 cardiomyoblasts through activation of GATA4. Our study emphasizes the role of RSK as a potential therapeutic target for indirectly alleviating hypertrophy induced by up regulation of NHE1 activity. Since RSKs are almost exclusively activated downstream of ERK1/2, therapeutic intervention by RSK inhibition is less likely to produce the side effects observed following inhibition of MEK and ERK1/2.

## Materials and Methods

All experimental procedures were in accordance with guidelines set out by the Institutional Biohazard Committee at Qatar University, Doha, Qatar.

### Materials

BCECF-AM was from Molecular Probes (Eugene, OR). BI-D1870 was purchased from the Division of Signal Transduction Therapy Unit, University of Dundee, Dundee, UK. EMD87580 (EMD) was a generous gift of Dr. N. Beier of Merck KGaA (Frankfurt, Germany). All other routine chemicals were of analytical grade and were purchased from BD Biosciences (San Jose, CA), Fisher Scientific (Ottawa, ON) or Sigma (St. Louis, MO). The primary antibodies used for western blotting were mouse monoclonal anti-HA-tag (6E2), rabbit polyclonal ERK1/2 (#9102), mouse monoclonal phospho-ERK1/2 (Thr^202^/Tyr^204^) (#9106), and rabbit polyclonal phospho-RSK (Ser^380^) (#9341), all from Cell Signaling Technology (Pickering, ON). The mouse monoclonal antibody against NHE1 was from BD Biosciences Pharmingen (San Diego, CA). Rabbit polyclonal RSK1 (C-21) (sc-231), goat polyclonal RSK2 (C-19) (sc-1430) and rabbit polyclonal p-GATA4 Ser^262^ (sc-32823) antibodies were from Santa Cruz Biotechnology (Santa Cruz, CA). Rabbit polyclonal antibodies for α-tubulin (ab4074) and GATA4 (ab-134057) were purchased from Abcam (Cambridge, MA). Secondary antibodies conjugated with peroxidase, goat-anti-mouse, goat-anti-rabbit and donkey-anti-goat were purchased from Jackson ImmunoResearch (West Grove, PA) or Abcam.

### Methods

#### Culturing of H9c2 cardiomyoblasts

H9c2 cardiomyoblasts, a clonal cell line derived from the embryonic BD1X rat heart tissue, were obtained from European Collections of Cell Cultures (ECACC) and cultured in DMEM/F12 1:1 culture media supplemented with 10% FBS and 1% penicillin/streptomycin (P/S) at 37°C in a humidified atmosphere (95% O_2_-5% CO_2_) [27]. Upon becoming confluent, cells were seeded at a density of 2.0 × 10^6^ cells per 35mm culture dishes containing the 10% FBS culture medium. In some experiments, H9c2 cardiomyoblasts were stimulated with 100μM PE in the presence or absence of 10μM BI-D1870 or EMD. Stock solutions of agonists/inhibitors were prepared at 1000× the working concentration and added directly to the tissue culture medium for the indicated time points. BI-D1870 was dissolved in DMSO, while PE and EMD were dissolved in water. For the PE treated experiments, H9c2 cardiomyoblasts were seeded in 35mm dishes for 24 hours in 10% FBS supplemented media. The media was then changed to serum-free maintenance medium for an additional 24 hours prior to treatment for the indicated time intervals were they were then subjected to lysing and immunoblotting.

#### Adenoviral preparation

The pAdTrack plasmids were used to engineer the NHE1 containing adenovirus as previously described [28]. The NHE1 plasmid also contained mutations in the Lys^641^, Arg^643^, Arg^645^ and Arg^647^ sites (to glutamic acid) that renders the protein active [29]. The RSK dominant-negative adenovirus (DN RSK) expresses a RSK protein with a mutation at Lys100Ala, which renders it catalytically inactive (a gift from Dr. M. Avkiran, King’s College, London, UK) [30, 31]. Both adenoviruses contained a hemagglutinin (HA) tag and the green fluorescent protein (GFP), which were used to confirm expression of the respective proteins.

#### Adenoviral infection of H9c2 cardiomyoblasts

H9c2 cardiomyoblasts were seeded in 35mm culture dishes containing the 10% FBS culture medium for 48 hours. H9c2 cardiomyoblasts were infected with the active NHE1 adenovirus in the presence or absence of the DN RSK adenovirus or an adenovirus containing GFP using a multiplicity of infection (MOI) of 50 for GFP and the active NHE1 adenoviruses respectively, while an MOI of 500 was used for the DN RSK encoding adenovirus (S1 Fig). Upon infection, the media was changed to DMEM-F12 HAM supplemented with 0.5% FBS and 1% P/S. Following infection, H9c2 cardiomyoblasts were incubated at 37°C in a humidified atmosphere (95% O_2_-5% CO_2_) incubator for an additional 48 hours prior to lysing, immunoblotting and all assays carried out.

#### Western blot analysis

H9c2 cardiomyoblasts were lysed 48 hours post infection using the radio-immunoprecipitation protein assay (RIPA) buffer as described earlier [27]. Briefly, cardiomyoblasts were lysed in 100 μL RIPA buffer in the presence of protease inhibitor cocktail buffer. Cell lysates were centrifuged at 14,000 rpm at 4°C and the supernatant containing the proteins were collected. Total amount of protein present in each sample was quantified using the DC protein assay kit from Biorad according to the manufacturer’s instructions. For the PE treatment experiments, cardiomyoblasts were sonicated in 1 mL of cell lysis buffer containing mM ((50 Na-pyrophosphate, 50 NaF, 50 NaCl, 5 EDTA, 5 EGTA, 0.1 sodium orthovandate, 10 Hepes) and 0.1% Triton X-100, 0.5 mM PMSF, 10 mg/mL leupeptin; pH 7.4)). For protein expression, 10–20 mg of protein was resolved on 9% SDS-PAGE and transferred on to nitrocellulose membranes. For phosphorylated protein expression, relative phosphorylation protein expression were obtained through normalizing the phosphorylated protein to total protein expression (pERK/ERK, pRSK/RSK, and pGATA4/GATA4). In some cases, the phosphorylated protein was normalized to α-tubulin, which was used as a loading control. Immunoreactive proteins were visualized using enhanced chemiluminescence (Amersham Biosciences) and imaged using the Alpha Innotech FluorChem Imager (R&D Systems).

#### Measurement of NHE1 activity

H9c2 cardiomyoblasts plated on a coverslip were loaded with 3μg/mL pH sensitive dye 2,7-bis(carboxyethyl)-5(6)-carboxyfluorescein acetoxymethyl ester (BCECF-AM). The change in H^+^ concentration was measured using a PTI Deltascan spectrofluorometer (Photon Technology International; London, Ontario). The excitation wavelengths were set at 502.5 nm and 440 nm and the emission wavelength was set at 528.7 nm. The cardiomyoblasts plated coverslip was initially maintained in a pre-warmed solution of Na^+^-normal buffer (mM (135 NaCl, 5 KCl, 1.8 CaCl_2_, 1 MgSO_4_, 5.5 Glucose, 10 HEPES) at 37°C. The coverslip plated with cardiomyoblasts was then subjected to 50 mM ammonium chloride to induce an acid load as previously described [31]. Following acidification, coverslips were placed in Na^+^ normal buffer to allow the cells to recover. Each coverslip was then equilibrated in a three-step pH calibration buffer solution containing 135 mM N-methyl-glucamine and KCl and adjusted to a pH of 8, 7 or 6. The pH calibration buffers were incubated with 10 mM nigericin, a K^+^ ionophore. The three-step pH calibration was used to generate a standard curve in which the fluorescence output measurements were converted into pH_i_ [9, 32]. The initial rate of recovery following an induced acid load was measured and used as an indicator of the NHE1 activity. For PE treated experiments, cells were first stimulated with the inhibitor (EMD-10μM) for 3 minutes at 37°C, followed by PE (100μM) for further 6 minutes. The treatment was maintained throughout the whole experiment, and the rate of recovery was calculated as mentioned previously [33].

#### Measurement of cell surface area

Surface area of infected cardiomyoblasts was measured 48 hours post infection. The average cell area of 50–70 randomly selected cells out of 6–7 experiments was taken. Cells were visualized with an inverted microscope equipped with a monochrome digitalized camera for the detection of fluorescence signals using 10X magnification. Cell surface area was determined using the AxioVision Imaging Software (Carl Zeiss Micro imaging, New York, NY).

#### Measurement of protein content

Protein content was measured as described previously [34]. H9c2 cardiomyoblasts in 35-mm dishes were washed twice in 1 x PBS and collected by trypsinization with 0.25% trypsin-EDTA solution (Invitrogen). The total number of cells was calculated using a Countess Cell Counting Chamber Slide (Invitrogen). Another set of infected H9c2 cardiomyoblasts in 35-mm dishes were lysed in RIPA buffer as described earlier [27]. Protein concentration of infected cardiomyoblasts lysed in RIPA buffer was measured using the DC protein assay kit (Biorad). Protein content was determined by calculating the total amount of protein (μg) in 10^6^ cells.

#### Expression of ANP mRNA using reverse transcription-polymerase chain reaction

RNA was extracted from cardiomyoblasts using the Total RNA Purification Kit (Norgen). Total RNA (1μg) was reverse transcribed into cDNA using SuperScript III First Strand Synthesis SuperMix (Invitrogen). 150-175ng of cDNA was amplified for ANP using sense 5′-CTGCTAGACCACCTGGAGGA-3′ and antisense 5′-AAGCTGTTGCAGCCTAGTCC-3′. β-actin cDNA was primed with sense 5′-GTTCCGATGCCCGAGGCTCT-3′ and antisense 5′-GCATTTGCGTGCACAGATGGA-3′(130) using 2x PCR Master Mix (Norgen) and used to normalize mRNA expression. Following an initial denaturation of 15 minutes at 95°C, the samples were denatured at 95°C for 10 seconds, annealed and extended at 60°C for 30 seconds for 35 cycles. A final extension of 72°C for 5 minutes was performed in order to ensure maximum recovery of products. ANP cDNA PCR products were then electrophoresed on 2% agarose gels stained with ethidium bromide. ANP and β-actin were detected at 320 and 147 bp respectively. ANP and β-actin were quantified using the Alpha Innotech FluorChem Imager. The changes in ANP mRNA levels were normalized to β-actin.

#### Statistics

All values expressed are compared to control ± SEM. Comparisons were performed by use of unpaired Student’s **t**-test and one-way ANOVA; a *P* value < 0.05 was considered statistically significant.

## Results

### Active NHE1 induces phosphorylation of RSK

Previous reports have demonstrated upregulation of NHE1 activity/expression in models of CH [35–38]. To elucidate the signaling pathway contributing to NHE1 dependent hypertrophy, an *in vitro* gain of function model was utilized. H9c2 cardiomyoblasts were infected with an adenoviral vector encoding for the active form of NHE1 using an MOI of 50 for 48 hours (S1 Fig). An enhanced green fluorescent protein (GFP)-expressing adenovirus under the control of the same promoter of the NHE1 adenovirus served as a control (S1 Fig). NHE1 activity was examined in H9c2 cardiomyoblasts infected with the active form of the NHE1 adenovirus and measured following an ammonium chloride pulse. NHE1 activity, indicative of the rate of recovery following an acid load, was significantly increased in H9c2 cardiomyoblasts infected with the active NHE1 adenovirus (885.6±156.96% (0.0119±0.0021 ΔpH_i_/min) vs. 100±33.99% (0.00134±0.00046 ΔpH_i_/min) control, *P*<0.05) (S2 Fig).

**Fig 1:**
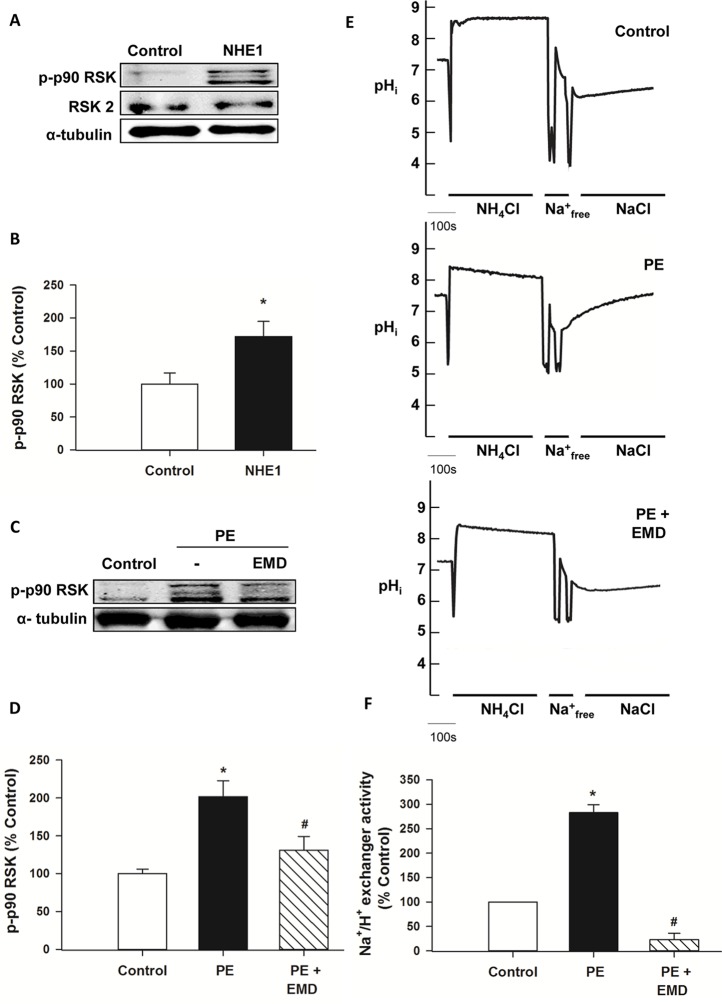
Active NHE1 increases phosphorylation of p90 ribosomal s6 kinase (RSK). **A**. Representative western blot of RSK protein expression in H9c2 cardiomyoblasts infected with GFP (control) or active NHE1 adenovirus. Immunoblotting was against *phosphorylated* RSK (90kDa), *total* RSK 2 (90kDa) or α- tubulin (50kDa); **B**. Quantification of experiments measuring the ratio of phosphorylated RSK to total RSK2 in H9c2 cardiomyoblasts infected with either the GFP (control) or active NHE1 adenovirus for 48 hours (n = 5). Results are expressed as % of control**±**SEM. *P<0.05 vs. control; **C.** Representative western blot of H9c2 cardiomyoblasts following treatment with PE in the absence or presence of EMD (NHE1 inhibitor). H9c2 cardiomyoblasts were either untreated (control), exposed to PE (100μM for 20 minutes), or pretreated with EMD (10μM, 30 minutes) prior to PE treatment for further 20 minutes. Immunoblotting was against *phosphorylated* RSK and α-tubulin; **D.** Quantification of experiments measuring relative levels of phosphorylation of RSK (normalized to tubulin) in H9c2 cardiomyoblasts stimulated with PE in the absence and presence of EMD pretreatment (n = 5). Results are expressed as % of control**±**SEM, *P<0.05 vs. control; ^#^ *P*<0.05 vs. PE; **E.** Representative NHE1 activity assay traces in H9c2 cardiomyoblasts for either untreated cells (control), treated with either PE (100μM, 6 minutes) alone or with EMD (10μM 3 minutes prior to PE treatment); **F**. Quantification of NHE activity of H9c2 cardiomyoblasts either untreated or treated with PE in the absence and presence of EMD (n = 4). Results are expressed as % of control**±**%SEM. *P< 0.05 vs. control; ^#^ *P*<0.05 vs. PE treated group.

Various stimuli induce activation and phosphorylation of RSK including ischemia [39], PE [40], ANG II [41, 42] and reactive oxygen species [43, 44], many of which are also known to activate NHE1. In order to further elucidate the signaling pathway of NHE1 induced hypertrophy, relative phosphorylation levels of RSK was examined by western blotting in H9c2 cardiomyoblasts infected with the active NHE1 adenovirus. RSK phosphorylation was significantly increased (172.17±22.68% vs. 100±16.8% control, *P*<0.05) in H9c2 cardiomyoblasts infected with the active NHE1 adenovirus (Figs. 1A and B). In order to further confirm our findings, H9c2 cardiomyoblasts were stimulated with PE, an α_1_ adrenoceptor agonist. H9c2 cardiomyoblasts stimulated with PE (100μM, 6 minutes) showed a significant increase in NHE1 activity (282.78±16.47% (0.002±0.0011 ΔpH_i_/min) of control, *P*<0.05), which was significantly decreased with prior treatment with EMD (10μM, 3 minutes) (22.94±13.26% (0.001±0.000925 ΔpH_i_/min) of control, *P*<0.05) (Figs. 1E and F). Exposure of H9c2 cardiomyoblasts to PE (100μM, 20 minutes) also increased phosphorylation of RSK (201.72±21.11% vs. 100±5.98% control, *P*<0.05), an effect that was significantly reduced upon pretreatment with 10μM EMD (130.89±18.49%, *P*<0.05) (Figs. 1C and D). Our findings establish that increased NHE1 activity results in activation and phosphorylation of RSK.

Examination of the phosphorylated expression of ERK1/2, an upstream target of RSK, following stimulation with PE reached a maximal response within 5 minutes (175.44±17.17% vs. 100±27.7% control, *P*<0.05) (S3 Fig). These effects were significantly reduced upon prior treatment with EMD (119.5±31.8% of control, *P*<0.05).

### Active NHE1 induces cardiomyocyte hypertrophy via RSK activation

**Fig 2:**
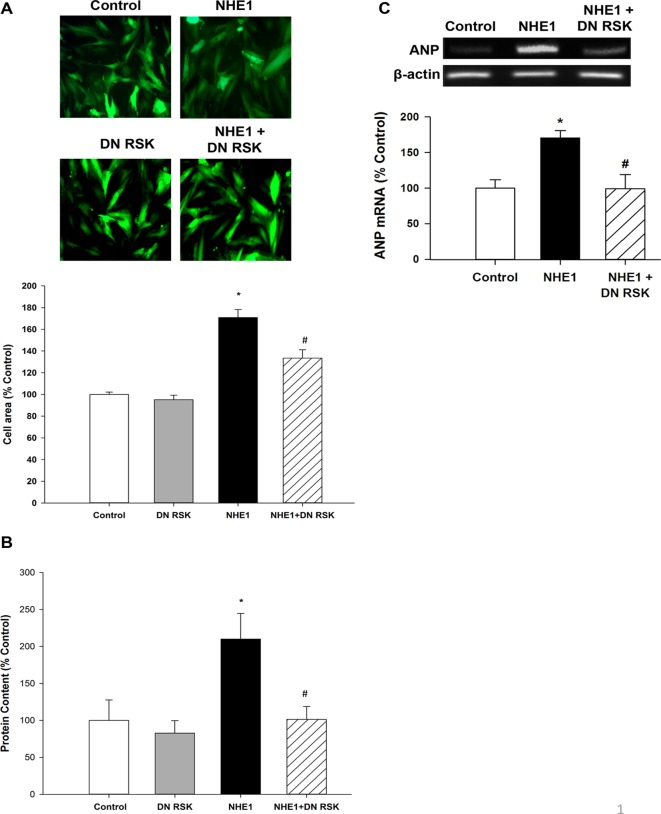
p90 Ribosomal S6 Kinase Mediates the NHE1 dependent hypertrophic response. Parameters of cardiomyocyte hypertrophy were analyzed in H9c2 cardiomyoblasts infected with either GFP (control), active NHE1, DN RSK or active NHE1 and DN RSK adenoviruses for 48 hours. **A.** Representative fluorescence microscopy images of H9c2 cardiomyoblasts infected with GFP (control), active NHE1, DN RSK or active NHE1 and DN RSK adenoviruses (*upper panel*). Quantification of surface cell area of H9c2 cardiomyoblasts infected with the GFP (control) active NHE1, DN RSK or active NHE1 and DN RSK adenoviruses (*lower panel*) (n = 6–7); **B**. Quantification of protein content of H9c2 cardiomyoblasts infected with the GFP (control), active NHE1, DN RSK or active NHE1 and DN RSK adenoviruses (n = 5–6); **C.** Representative agarose DNA gel showing ANP and β-actin. H9c2 cardiomyoblasts were either left untreated (control), or infected with active NHE1 or active NHE1 and DN RSK adenoviruses for 48 hours (*upper panel*). RNA extracted from H9c2 cardiomyoblasts was reverse transcribed and amplified using ANP and β-actin primers, respectively. Quantification of experiments measuring ANP mRNA expression normalized to β-actin in H9c2 cardiomyoblasts which were either left untreated (control) or infected with active NHE1 or active NHE1 and DN RSK adenoviruses for 48 hours (*lower panel*) (n = 7). Results are expressed as % of control**±**SEM *P<0.05 vs. control; ^#^ *P*<0.05 vs. NHE1 infected group.

Although RSK has been suggested to mediate CH [21, 23, 45], whether RSK contributes to hypertrophy induced by elevated expression/activity of NHE1 has not been shown. To identify the role of RSK in the NHE1 mediated hypertrophic effect, NHE1 expression/activity was induced in cardiomyoblasts expressing the DN RSK adenovirus, which blocked the RSK kinase domain activity. Expression of active NHE1 in H9c2 cardiomyoblasts caused a significant increase in cell area and protein content (170.85±7.38%, *P*<0.05 and 210.1±34.47%, *P*<0.05 of control, respectively) (Figs. 2A and B). These findings suggest that enhanced expression of active NHE1 induces hypertrophy in H9c2 cardiomyoblasts. H9c2 cardiomyoblasts expressing both active NHE1 and DN RSK demonstrated a significant decrease in cell area (133.48±7.72%, *P*<0.05) and protein content (101.28±17.45%, *P*<0.05) compared to H9c2 cardiomyoblasts infected with the active NHE1 adenovirus alone (Figs. 2A and B). We also investigated the effect of active NHE1 on ANP mRNA expression, another marker of hypertrophy [46]. ANP mRNA expression in H9c2 cardiomyoblasts expressing active NHE1 significantly increased (176.6±19.38% vs. 100±11.75% control, *P*<0.05) (Fig. 2C). Co-infection of the active form of the NHE1 adenovirus with the DN RSK adenovirus demonstrated a significant decrease in ANP mRNA expression compared to the NHE1 infected group alone (122.9±29.64%, *P*<0.05). H9c2 cardiomyoblasts infected with the DN RSK adenovirus alone showed no significant changes in cell surface area (95.21±4.51% of control) and protein content (82.71±16.9% of control) (Figs. 2A and B).

### Effect of RSK inhibition on NHE1 protein expression and activity

**Fig 3:**
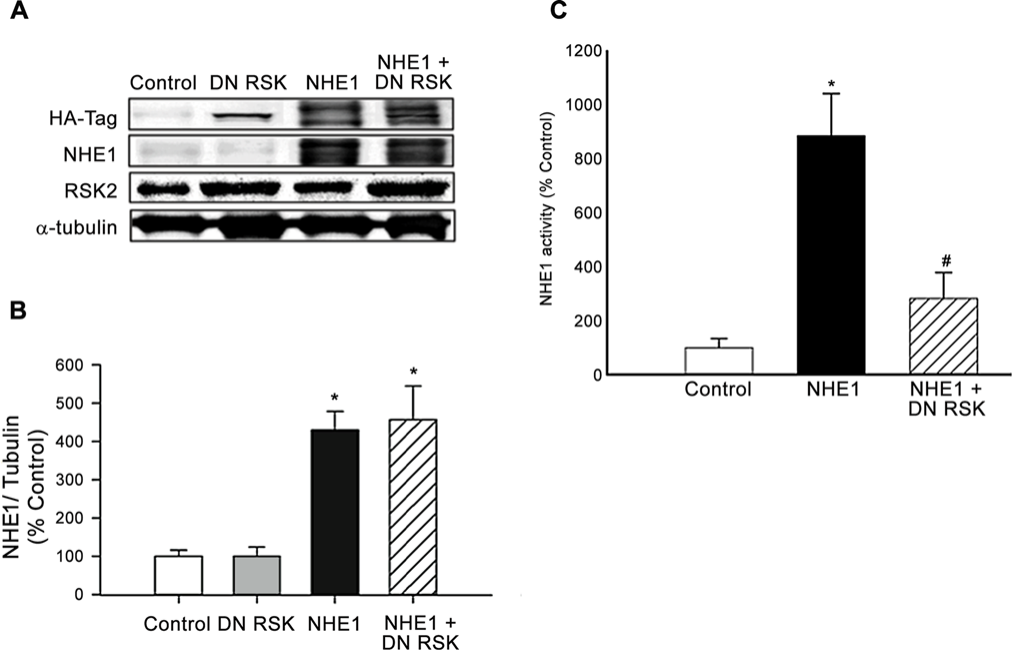
NHE1 protein expression and activity in H9c2 cardiomyoblasts infected with active NHE1 and DN RSK. Protein expression levels of total NHE1 protein in H9c2 cardiomyoblasts infected with either GFP (control), DN RSK, active NHE1 or active NHE1 and DN RSK adenoviruses. **A.** Representative western blot of H9c2 cardiomyoblasts infected with GFP (control), DN RSK, active NHE1 or active NHE1 and DN RSK adenoviruses for 48 hours. Immunoblotting was against HA-tag for the expression of *exogenous* RSK and NHE1, *total* NHE1 (90-110kDa), RSK2 (90kDa) or α-tubulin (50kDa); **B.** Quantification of experiments measuring *total* NHE1 protein expression, normalized to α-tubulin in H9c2 cardiomyoblasts infected with GFP (control), DN RSK, active NHE or active NHE1 and DN RSK adenoviruses for 48 hours (n = 5); **C**. Quantification of NHE1 activity of H9c2 cardiomyoblasts infected with GFP (control), active NHE1 or active NHE1 and DN RSK adenoviruses for 48 hours. NHE activity was calculated as the rate of recovery following induction of acid load (n = 5–6). All Results are expressed as % of control (GFP)±SEM. *P<0.05 vs. control, ^#^ *P*<0.01 vs. NHE1 infected group.

RSK has been demonstrated to be a primary regulator of NHE1 activity through phosphorylation of Ser^703^ at the NHE1 C-terminal, and to facilitating the binding of 14-3-3 protein to the phosphorylated Ser^703^ residue [20, 22, 47]. However, the possible role of NHE1 upstream of RSK in the setting of cardiac hypertrophy has so far been unknown. To further elucidate whether DN RSK effects NHE1 protein expression, we examined both exogenous and endogenous NHE1 protein expression in H9c2 cardiomyoblasts infected with either the GFP (control), active form of NHE1 or DN RSK in the presence or absence of the NHE1 adenovirus (Fig. 3A). NHE1 protein expression was significantly increased in H9c2 cardiomyoblasts infected with NHE1 (430.05±48.63% vs. 100±15.8% control, *P*<0.05) and NHE1 with DN RSK (410.61±122.43% of control, *P*<0.05). However, no significant difference in NHE1 protein expression was seen when comparing H9c2 cardiomyoblasts infected with NHE1 in the absence (430.05±48.63% of control) or the presence of DN RSK (410.6±122.43% of control) (Fig. 3B). Although no significant changes were observed in NHE1 protein expression, H9c2 cardiomyoblasts co-infected with the active form of the NHE1 adenovirus and the DN RSK adenovirus resulted in a significant decrease in NHE1 activity (281.64± 96.71% (0.00379±0.0013 ΔpH_i_/min of NHE1 alone; *P*<0.01) (Fig. 3C).

### NHE1 induces GATA4 phosphorylation via RSK

**Fig 4:**
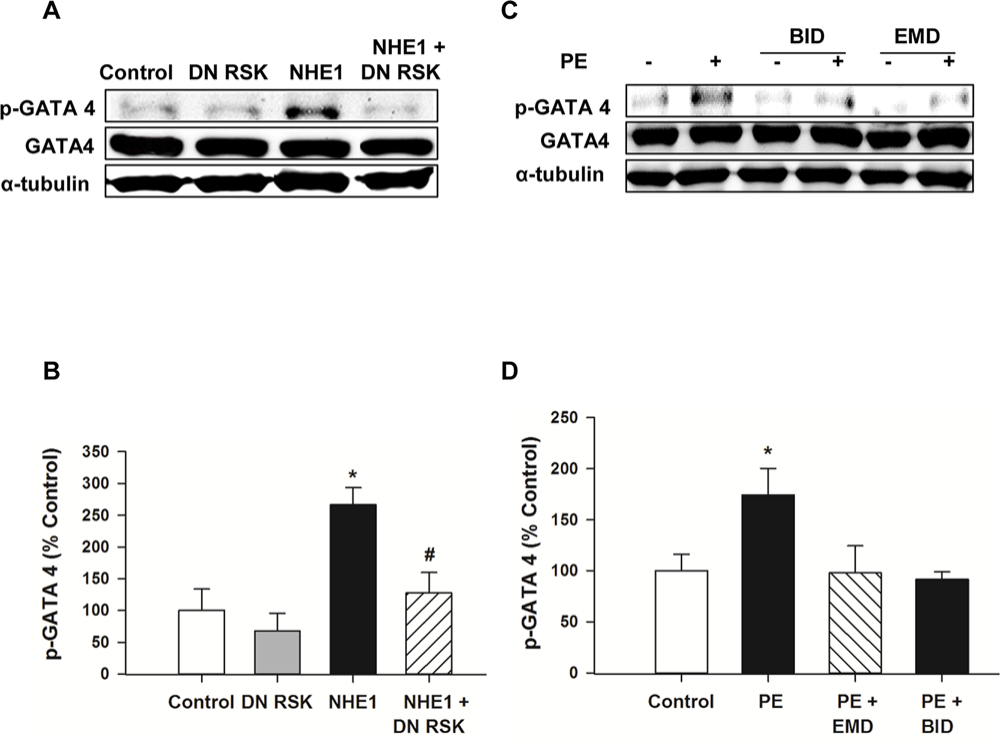
Inhibition of RSK attenuated GATA4 phosphorylation mediated by activation of NHE1. **A**. Representative western blot for H9c2 cardiomyoblasts infected with either GFP (control), DN RSK, active NHE1 or active NHE1 and DN RSK adenoviruses for 48 hours. Immunoblotting was against *phosphorylated* GATA4 (46kDa), *total* GATA4 (50kDA) or α-tubulin (50kDa); **B**. Quantification of experiments measuring the ratio of phosphorylated GATA4 to total GATA4 in H9c2 cardiomyoblasts infected with either GFP (control), DN RSK, active NHE1 or active NHE1 and DN RSK adenoviruses for 48 hours. Results are expressed as % of control**±**SEM (n = 5). *P<0.05 vs. control. ^*#*^ *P*<0.05 vs. NHE1 infected group. RSK or NHE1 inhibition attenuates PE induced GATA4 phosphorylation. H9c2 cardiomyoblasts were either untreated (control) or treated with PE (100μM for 20 minutes) in the presence and absence of either BID 1870 (10μM), or EMD (10μM) added 30 minutes prior to PE treatment (100μM); **C.** Representative western blot showing *phosphorylated* GATA4, *total* GATA4 and α-tubulin; **D**. Quantification of relative phosphorylation levels of GATA4. Results are expressed as % of control**±**SEM. *P <0.05vs. control, ^*#*^ *P*<0.05 vs. NHE1 infected group.

GATA binding protein 4 (GATA4) is a transcription factor well known to be implicated in the hypertrophic stimuli. GATA4 can be phosphorylated by MAPKs in response to hypertrophic stimuli such as PE and endothelin-1 at Ser^105^, leading to increased transcriptional activity [48, 49]. In a recent study, inhibition of RSK in NRVMs inhibited PE induced phosphorylation of GATA4 at Ser^261^, ultimately leading to repression of fetal cardiac genes [26]. Similarly, previous reports have established that increased NHE1 activity leads to activation of the calcineurin/ NFAT signaling pathway [50, 51], which was associated with enhanced GATA4 activation and nuclear translocation [51]. We have examined whether RSK modulates NHE1 induced hypertrophy via regulation of GATA4. GATA4 phosphorylation levels were significantly increased in NHE1 infected H9c2 cardiomyoblasts (266.56±26.69% vs. 100±33.8% control, *P*<0.01), which was significantly reduced in the presence of the DN RSK adenovirus (127.31±32.9% of control, *P*<0.05) compared to the NHE1 infected group (266.56±26.69% of control) (Fig. 4A and 4B). Similar findings were obtained with PE stimulation (100μM, 20 minutes); a significant increase in GATA4 relative phosphorylation (174.39±25.9% of control, *P*<0.05) (Fig. 4C). This effect was reversed upon prior treatment with either BI-D1870 (91.5±7.3%, *P*<0.05) or EMD (97.7±26.7%, *P*<0.05) compared to PE alone (Fig. 4D). These results indicate for the first time that the key and novel role for RSK in the reduction of GATA4 phosphorylation and activation induced by active NHE1.

## Discussion

The expression of constitutively active NHE1 and RSK has both been shown to individually contribute to the development and progression of CH [50, 52, 53]. Earlier studies have confirmed the central role of NHE1 in the regulation of MAPK activity in cardiac hypertrophy [54, 55]. However, no reports have directly investigated whether expression of active NHE1 induces RSK and how RSK contributes to the NHE1-mediated hypertrophic response in cardiomyocytes. The primary focus of this study was to investigate the potential contribution of RSK activation in mediating active NHE1 induced hypertrophy in cardiomyocytes and to further assess the underlying mechanism.

### NHE1 and MAPK

Previous reports have demonstrated that the anti-hypertrophic effect of NHE1 inhibition (with EMD 87580) in PE treated NRVMs was found to be associated with prevention of MAPK activation [50]. More specifically, NHE1 inhibition has been shown to reduce stretch induced ERK1/2 activation in NRVMs [54] as well as activation of ERK1/2 in rat aortic smooth muscle cells following stimulation with ANG II or 5-hydroxytryptamine [18]. In our study, a transient increase in ERK1/2 phosphorylation was observed upon treatment of H9c2 cardiomyoblasts with PE for 5 minutes (S3 Fig), which was inhibited with prior treatment with EMD. In agreement with our study, Haworth RS et al has reported that sustained acidosis increases the activity of ERK, with peak activation after > 3 minutes [56]. The ability of NHE1 inhibition to attenuate ERK1/2 phosphorylation at 5 minutes is in agreement with a previous study showing that hyperglycemia-induced activation of ERK1/2 in NRVMs can be prevented through NHE1 inhibition cariporide [57]. Although ERK1/2 are central mediators of cardiac hypertrophy, direct inhibition of ERK1/2 has lead to exacerbated cardiomyocyte death and impaired heart function [58]. Since RSK is the main downstream effector of ERK; it provided an attractive target for therapeutic intervention to avoid the deleterious side effects of inhibiting the upstream regulator, ERK1/2 [21]. In our study, we focused on phosphorylation of RSK and its involvement in mediating hypertrophy induced by active NHE1.

RSK consists of three principle isoforms (RSK 1, 2 and 3). These isoforms all bind to and are activated by ERK1/2. An additional isoform, RSK4 has been also identified. Yet, recent evidence suggests that it behaves in a typical manner is terms of its expression and function [22]. In our study, we focused on the RSK 2—DN RSK adenovirus. However, our conclusion does not exclude the role of other isoforms since the approach used to interfere with RSK activity was either the RSK 2—DN RSK or through a pharmacological inhibitor (BID 1870) [59], both of which could not fully identify the potential role of other RSK isoforms.

Previous studies have revealed that cellular RSK activity is increased in response to multiple hypertrophic stimuli, such as catecholamines (via α1-adrenoceptors) [60–62], ANG II [41], endothelin-1 [62], oxidative stress [63] and ischemia/reperfusion [64]. Furthermore, RSK expression and activity appear to be increased in animal models of heart failure and in failing human myocardium [65, 66]. In NRVMs, nuclear fraction of RSK2 isoform was upregulated in response to hypertrophic stimuli of endothelin-1, ANG II and PE; where they played a significant role in early gene regulation in cardiomyocytes [67]. Previous reports have demonstrated that RSK phosphorylates NHE1 at Ser^703^ [20] and thereby mediates increased cardiac sarcolemmal NHE1 activity. In our study, we demonstrated that expression of active NHE1 in H9c2 cardiomyoblasts increases the phosphorylation levels of RSK upon infection of H9c2 cardiomyoblasts with the active NHE1 adenovirus, which was associated with hypertrophy. In addition, we demonstrated that NHE1 inhibition attenuated RSK phosphorylation stimulated with PE, suggesting for the first time that RSK phosphorylation is regulated by NHE1. Parameters of hypertrophy were significantly regressed upon concomitant infection of H9c2 cardiomyoblasts with active NHE1 and the DN RSK adenovirus, suggesting the involvement of RSK in the NHE1 induced hypertrophic response. The reduction of NHE1 activity upon co-infection of the NHE1 and DN RSK adenovirus suggests that a feedback loop exists and may in part be due to the inability of RSK to phosphorylate and activate NHE1.

### NHE1 and Cellular pH

The functional coupling of NHE1 to the Na^+^/Ca^2+^ exchanger increases sarcoplasmic-reticular Ca^2+^ loading and release during intracellular acidosis, conditions associated with ischaemia-reperfusion and some forms of maladaptive hypertrophy [68]. In primary cultures of cardiomyocytes, NHE1 has been shown to be elevated following the induction of transient as well as sustained acidosis in an effort to drive the reduction in cytosolic pH [28]. Sustained intracellular acidosis has also previously been demonstrated to activate both ERK and RSK [56], with peak activation achieved after >3 minutes. On the contrary, in our study, peak activation of RSK following PE stimulation occurred at 20 minutes, similar to the time needed to phosphorylate RSK by oxidative stress (H_2_0_2_) [69]. Despite the importance of NHE1 activity in driving a reduction in cytosolic pH, a previous report has demonstrated that the mechanism driving ERK1/2 [19], is not dependent on the ions transported via NHE1 [17]. Whether cellular pH contributes to the mechanism driving RSK phosphorylation downstream of NHE1 and contributing to the hypertrophic response remains to be identified. Future studies investigating the role of oxidative stress in mediating the NHE1-RSK cascade is also important, particularly since a previous report has demonstrated that the anti-hypertrophic effects of EMD are mediated by the reduction of ROS production, a mediator involved in RSK activation [70].

### Possible role of GATA4 in NHE1 induced hypertrophy

**Fig 5:**
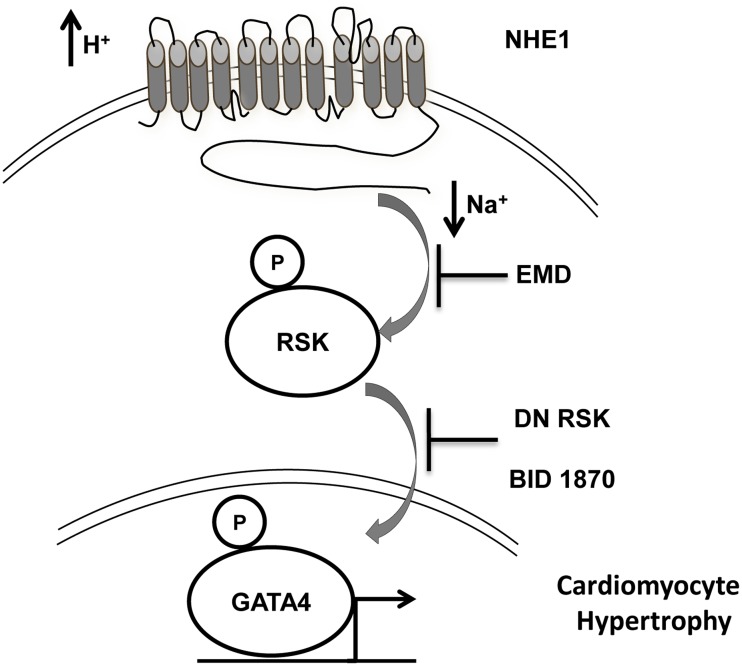
NHE1 induces phosphorylation and activation of RSK which would result in GATA4 phosphorylation. Leading to activation of transcription machinery and modulation of the hypertrophic pathway. Inhibition of RSK attenuates the NHE1 induced cardiomyocyte hypertrophic response.

GATA4 is an important transcription factor mediating cardiac hypertrophy. GATA4 is considered a key transcriptional regulator of numerous peptides synthesized in the heart, including ANP and BNP [71]. The activity of GATA4 is modulated through direct interactions and post translational modifications including phosphorylation, acetylation and soumylations [72]. GATA4 can be phosphorylated by MAPKs in response to hypertrophic stimuli such as PE and endothelin-1, leading to increased transcriptional activity [48, 49]. In a recent study, inhibition of RSK in NRVMs inhibited PE induced phosphorylation of GATA4 at Ser^261^, ultimately leading to repression of fetal cardiac genes [26]. Upon stimulation, RSK 2 isoform induce nuclear localization and activation of NFATc4 transcription activation complex [73]. Previous findings have established that increased NHE1 activity lead to activation of calcineurin/ NFAT signaling pathway [50, 51], which was associated with enhanced GATA4 activation and nuclear translocation [51]. In our model, the increase in GATA4 phosphorylation observed in response to expression of active NHE1 was significantly abrogated upon concomitant transfection with DN RSK. Similarly, we found a significant increase in phosphorylation of GATA4 following treatment with PE (20 minutes), which was significantly reduced with prior treatment with either RSK or NHE1 inhibitors. All these findings suggest that RSK lies down stream of NHE1, and regulates NHE1 induced cardiomyocyte hypertrophy through modulation of GATA4 phosphorylation (Fig. 5).

Our results show for the first time that NHE1 induced cardiomyocyte hypertrophy can be attributed to phosphorylation and activation of GATA4 secondary to active NHE1 induction of RSK activation.

## Supporting Information

S1 Fig Multiplicity of infection (MOI) and duration of NHE1 and DN RSK adenoviral infection in H9c2 cardiomyoblasts. Immunoblotting was against the HA-tag for *exogenous* NHE1 (90-110kDa) and RSK (90kDa) protein expression. **A.** Representative fluorescence microscopy images of H9c2 cardiomyoblasts infected with control (GFP), active NHE1 or DN RSK adenoviruses 48 hours post infection. An MOI of 20, 30 or 50 was used for GFP (control) and NHE1 adenoviruses, while an MOI of 200, 500 or 1000 was used for the DN RSK adenovirus; **B**. Representative western blot of H9c2 cardiomyoblasts infected with control (GFP) or active NHE1 (20, 30 or 50 MOIs) or DN RSK adenovirus (200, 500 or 1000 MOIs) 48 hours post infection (n = 3).
(TIF)

S2 Fig Na^+^/H^+^ exchanger activity (NHE) is increased upon infection with the NHE1 adenovirus in H9c2 cardiomyoblasts. NHE activity was calculated as the rate of recovery following induction of acid load. **A**. Representative traces of NHE1 activity assays in H9c2 cardiomyoblasts infected with GFP (control) or active NHE1 48 hours post infection; **B**. Quantification of NHE1 activity (n = 5–6). Results are expressed as % of control (GFP)±SEM. *P<0.05 vs. control.
(TIF)

S3 Fig Phenylephrine induced phosphorylation of extracellular regulated kinase (ERK). **A.** Representative western blot for H9c2 cardiomyoblasts treated with either vehicle, or with phenylephrine (PE) (100μM) in the absence and presence of EMD (10μM) added 30 minutes prior to PE (100μM) treatment for 5 minutes. Immunoblotting was against phosphorylated ERK and total ERK. The phosphorylated ERK was normalized to total ERK; **B.** Quantification of relative levels of phosphorylated ERK (n = 4). Results are expressed as % of control**±**SEM. *P<0.05 vs. control. ^#^ *P*<0.05 vs. PE treatment alone.
(TIF)
