# Supplementary figures and images for: Na+/H+ Exchanger Isoform 1 Induced Cardiomyocyte Hypertrophy Involves Activation of p90 Ribosomal S6 Kinase
Source: PLoS One. 2015 Apr 1;10(4):e0122230. doi: 10.1371/journal.pone.0122230 (PMC4382094; doi:10.1371/journal.pone.0122230)

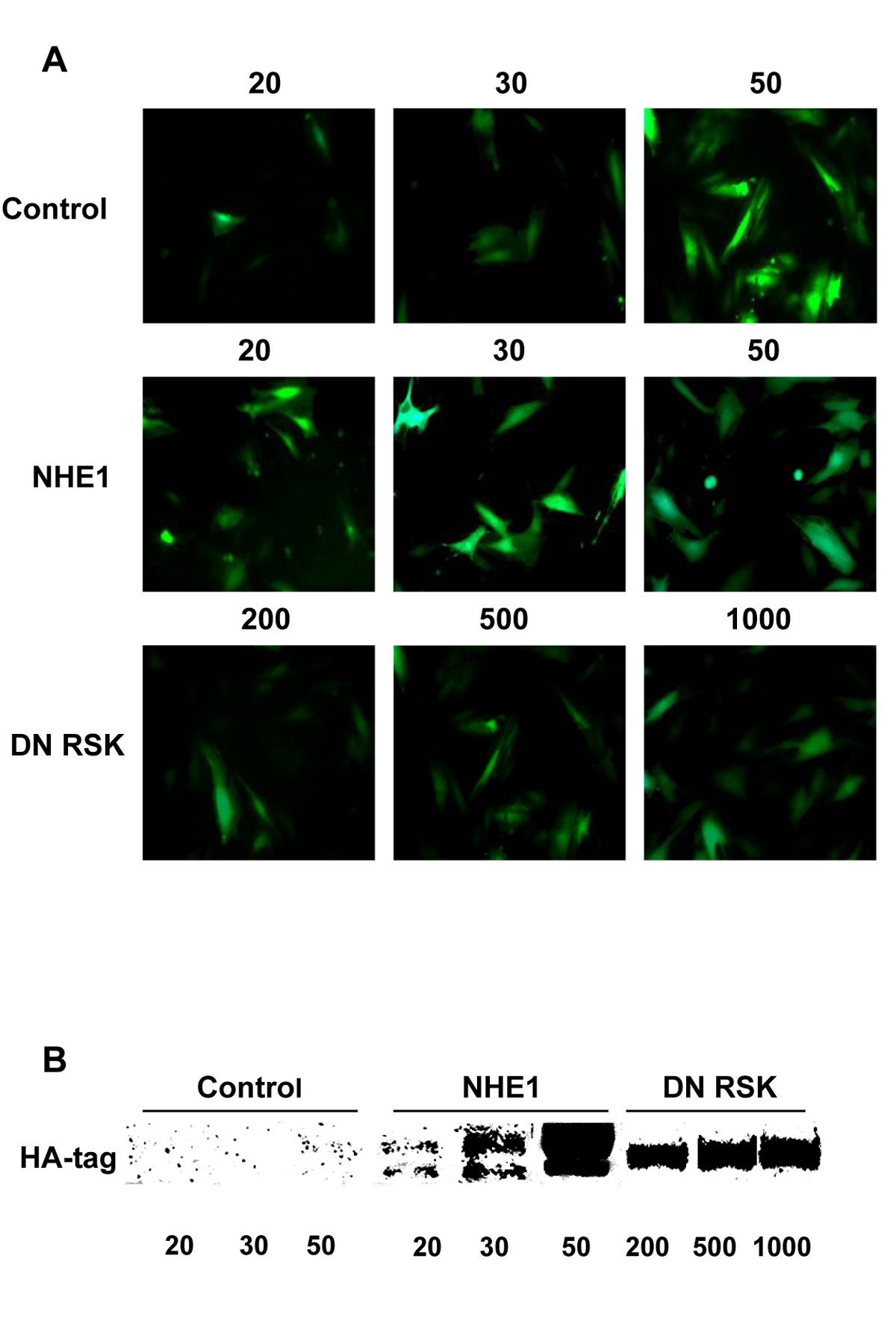

Supplement: S1 Fig — Immunoblotting was against the HA-tag for exogenous NHE1 (90-110kDa) and RSK (90kDa) protein expression. A. Representative fluorescence microscopy images of H9c2 cardiomyoblasts infected with control (GFP), active NHE1 or DN RSK adenoviruses 48 hours post infection. An MOI of 20, 30 or 50 was used for GFP (control) and NHE1 adenoviruses, while an MOI of 200, 500 or 1000 was used for the DN RSK adenovirus; B. Representative western blot of H9c2 cardiomyoblasts infected with control (GFP) or active NHE1 (20, 30 or 50 MOIs) or DN RSK adenovirus (200, 500 or 1000 MOIs) 48 hours post infection (n = 3). (TIF) [file pone.0122230.s001.tif]

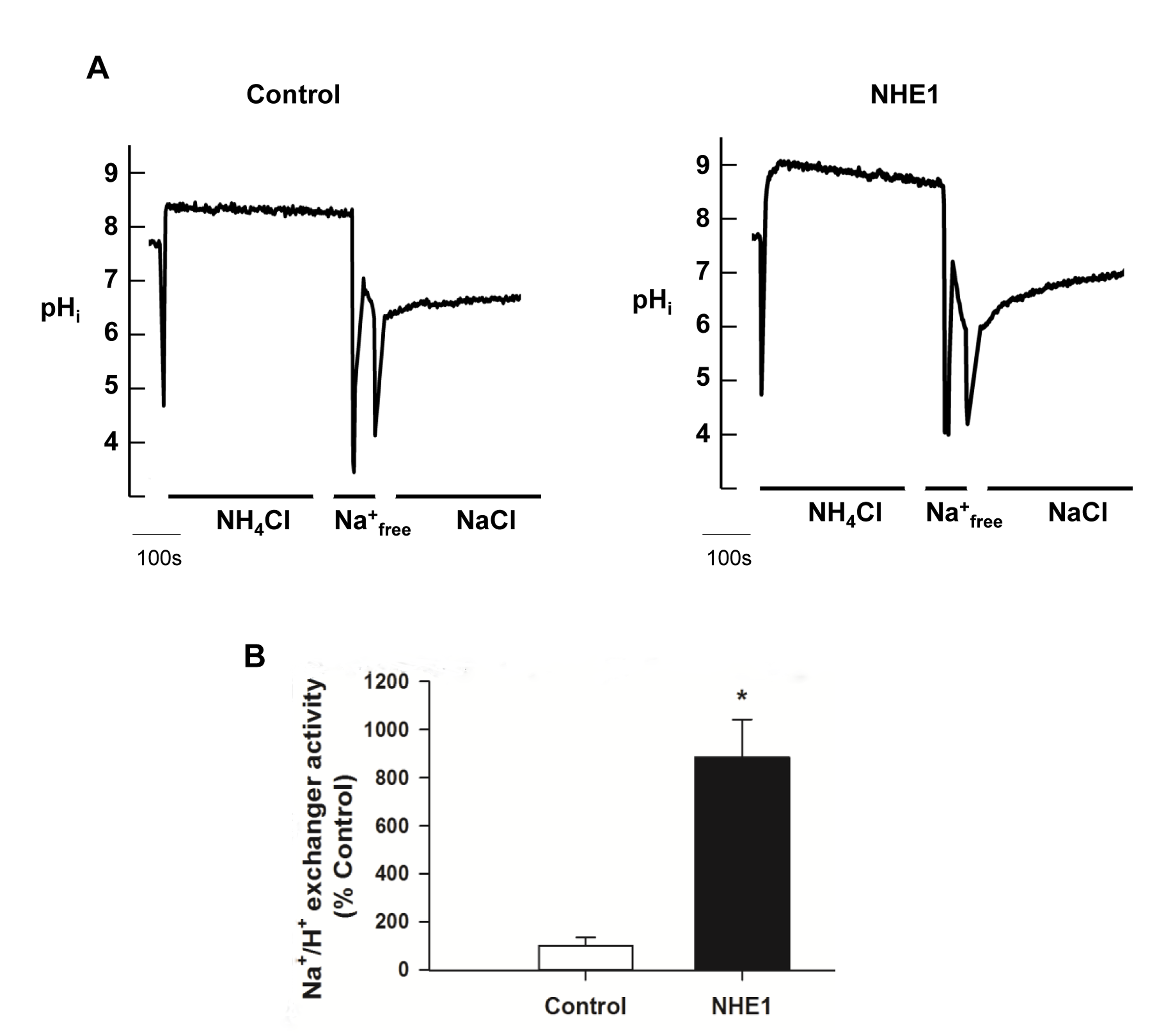

Supplement: S2 Fig — NHE activity was calculated as the rate of recovery following induction of acid load. A. Representative traces of NHE1 activity assays in H9c2 cardiomyoblasts infected with GFP (control) or active NHE1 48 hours post infection; B. Quantification of NHE1 activity (n = 5–6). Results are expressed as % of control (GFP)±SEM. *P<0.05 vs. control. (TIF) [file pone.0122230.s002.tif]

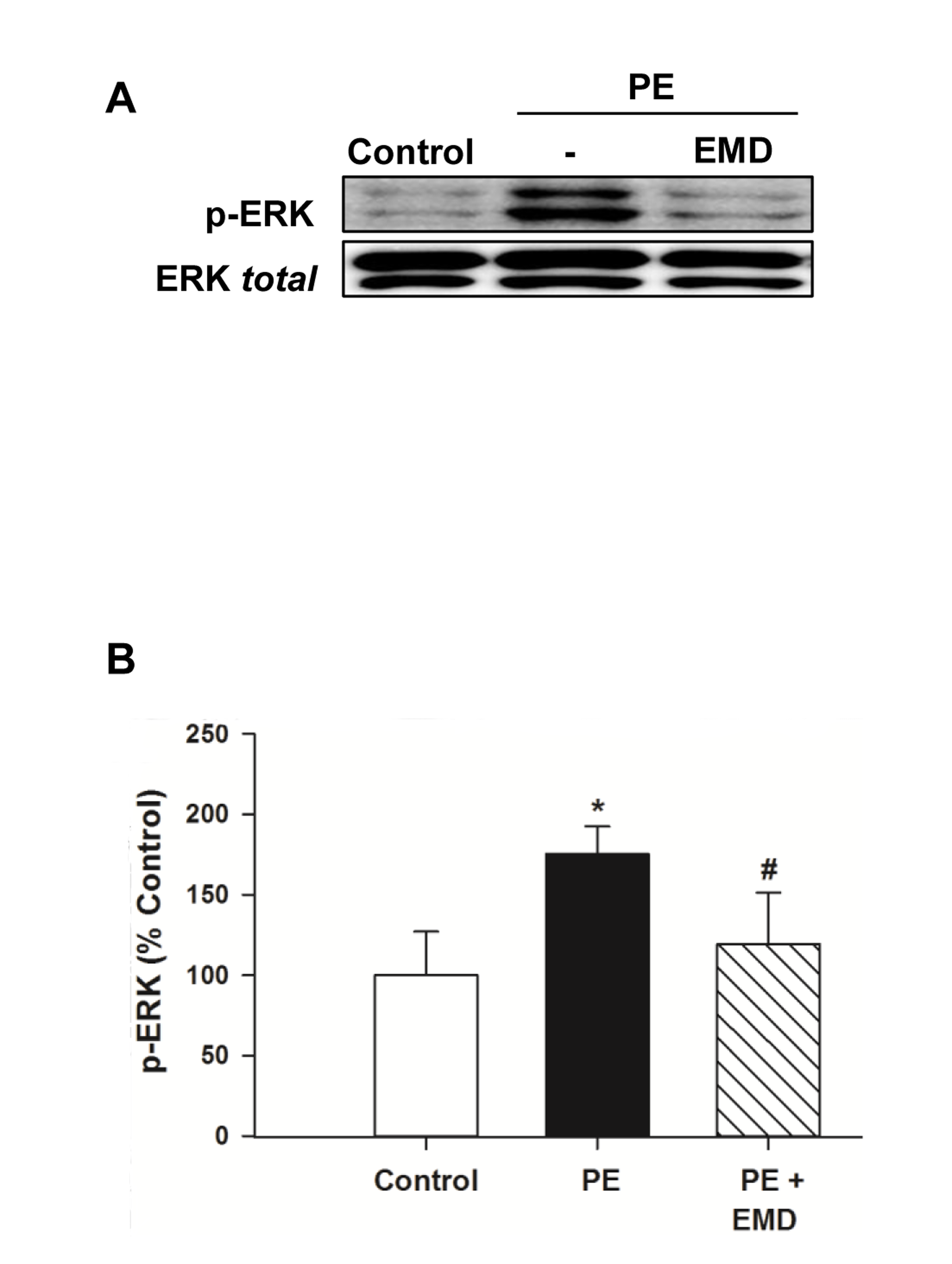

Supplement: S3 Fig — A. Representative western blot for H9c2 cardiomyoblasts treated with either vehicle, or with phenylephrine (PE) (100μM) in the absence and presence of EMD (10μM) added 30 minutes prior to PE (100μM) treatment for 5 minutes. Immunoblotting was against phosphorylated ERK and total ERK. The phosphorylated ERK was normalized to total ERK; B. Quantification of relative levels of phosphorylated ERK (n = 4). Results are expressed as % of control±SEM. *P<0.05 vs. control. # P<0.05 vs. PE treatment alone. (TIF) [file pone.0122230.s003.tif]
